# Testing day: The effects of processing bias induced by Navon stimuli on the strength of the Müller-Lyer illusion

**DOI:** 10.2478/v10053-008-0151-8

**Published:** 2014-02-20

**Authors:** Matthew E. Mundy

**Affiliations:** School of Psychology and Psychiatry, Monash University, Australia

**Keywords:** Müller-Lyer, processing bias, global, local, Navon stimuli

## Abstract

Explanations for the cognitive basis of the Müller-Lyer illusion are still frustratingly mixed. To date, Day’s (1989) theory of perceptual compromise has received little empirical attention. In this study, we examine the merit of Day’s hypothesis for the Müller-Lyer illusion by biasing participants toward global or local visual processing through exposure to Navon (1977) stimuli, which are known to alter processing level preference for a short time. Participants (*N* = 306) were randomly allocated to global, local, or control conditions. Those in global or local conditions were exposed to Navon stimuli for 5 min and participants were required to report on the global or local stimulus features, respectively. Subsequently, participants completed a computerized Müller-Lyer experiment where they adjusted the length of a line to match an illusory-figure. The illusion was significantly stronger for participants with a global bias, and significantly weaker for those with a local bias, compared with the control condition. These findings provide empirical support for Day’s “conflicting cues” theory of perceptual compromise in the Müller-Lyer illusion.

## Introduction

**Figure 1.**
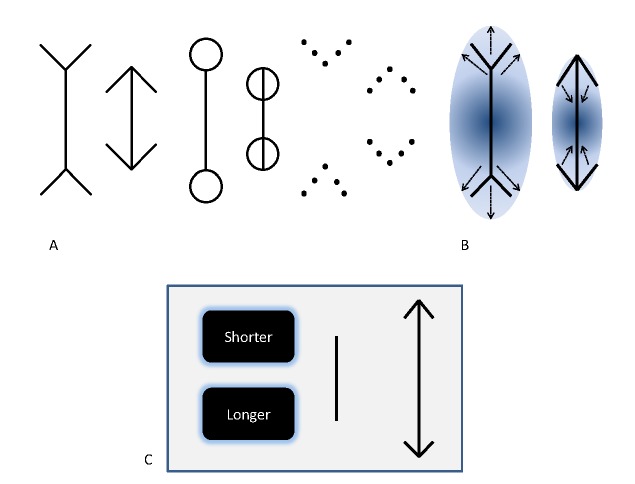
Panel A. Variants of the Müller-Lyer illusion including the original (left), dumbbell (centre) and dot type (right). Panel B. Day’s (1989) theory of perceptual compromise suggests that the overall size of the figure and distance between features influences our perception of the central line: A conflict exists between the global cue for overall size and the local requirement to judge line length. Panel C. An example of a single trial, where participants were asked adjust the vertical line (with no fins) to match the length of the line of the illusory figure. The computer mouse is used click the appropriate button to make the adjustable line smaller or larger.

The Müller-Lyer (1889) illusion consists of two identical lines that appear different in length, due to arrowheads or arrowtails fixed at the apexes of both lines (Panel A of Figure 1). The line with the arrowheads (“fins-in”) is perceived as being shorter than the line with the arrowtails (“fins-out”). Numerous theories and experimental paradigms have been explored in an attempt to explain this illusion, such as misapplied size constancy (Gregory, 1963), variations of angle degree and length of arrowheads (Dewer, 1967; Pressey & Martin, 1990; Restle & Decker, 1977), and central-tendency-effects (Pressey, 1967; see also Bertulis & Bulatov, 2001; Woloszyn, 2010).

A longstanding explanation for this illusion is that of “misapplied size constancy” (Gregory, 1963). This theory suggests that size constancy applied to objects in three-dimensional space is misapplied to two-dimensional figures due to learned depth cue confusion (see also McGraw & Stanford, 1994). The effect is typically demonstrated by comparisons between an interior room corner and an exterior building corner. Researchers have challenged this hypothesis, however, arguing that it cannot explain the illusion’s persistence in dumbbell or dot presentations, where the termini of the lines cannot be conceptualised as corners or edges in three dimensions (e.g., Day, 1989; Lamy, Segal, & Ruderman, 2006; Woloszyn, 2010; see Panel A of Figure 1). Furthermore, individuals whom have little experience of linear architecture are still susceptible to the illusion; albeit to a lesser extent (Ahluwalia, 1978). Thus, there is still no consensus of explanation within the literature, particularly as many theories fail to explain various modifications of the basic illusion (see Robinson, 1998).

Day (1989) proposed a theory of “perceptual compromise” to explain visual illusions like Müller-Lyer’s. He suggested that the Müller-Lyer illusion arises due to conflicting cues, where a compromise exists between the true lengths of the lines and interapical distances (local features) and the lengths of the complete figures (global features). Therefore, the line with arrowtails at its apexes, or fins-out, appears longer than the line with arrowheads (or fins-in) since the fins-out figure is larger overall and the distance between the a pices of the fins is greater (see Panel B of Figure 1). Although Day’s mechanism is less specific regarding how the line with arrowheads at its apexes (or fins-in) might appear shorter, it would seem a natural extension of his theory to suggest that a shorter interapical distance would result in a reduction in line estimation, as the greater global feature is now concentrated in the centre of the figure (see Panel B of Figure 1). Thus, there appear to be global size differences between the two examples of the illusion, even though local features (line length) are the same. It is suggested, therefore, that a perceptual compromise is sought between this conflicting information, resulting in the illusion. Unlike some competing theories, perceptual compromise successfully accounts for illusions created by variations of the original Müller-Lyer figure. Day’s theory has been proposed as an explanation for the following illusions: the Poggendorff illusion (Day & Kasperczyk, 1985), the Morinaga illusion (Day, 1989), arc and chevron illusions (Day, Jee, & Duffy, 1989), and the Bourdon illusion (Day, 1990). Other authors have described similar mechanisms which rely on mismatch, assimilation, or compromise between local features and global or gestalt figure features (e.g., Lamy et al., 2006; Morgan & Glennerster, 1991; Pressey, 1967, 1971; Woloszyn, 2010). However, to the author’s knowledge, Day’s theory, or those related to it, have not seen *direct* empirical testing with respect to Müller-Lyer and illusion magnitude.

Interpreting Day’s (1989) theory, if one were to create a bias in an observer’s attention towards global processing then the magnitude of the illusion should be increased. If a participant is focusing on gestalt or global “wholes” then a compromise between the conflicting global and local cues present in the figure will be biased toward the global (overall stimulus size) cue. Conversely, if an attention bias were to exist towards local processing, thus reducing the influence of the fins, then the magnitude of the Müller-Lyer illusion should decrease, since the observer will attend more closely to the local (individual line length) cue.

**Figure 2.**
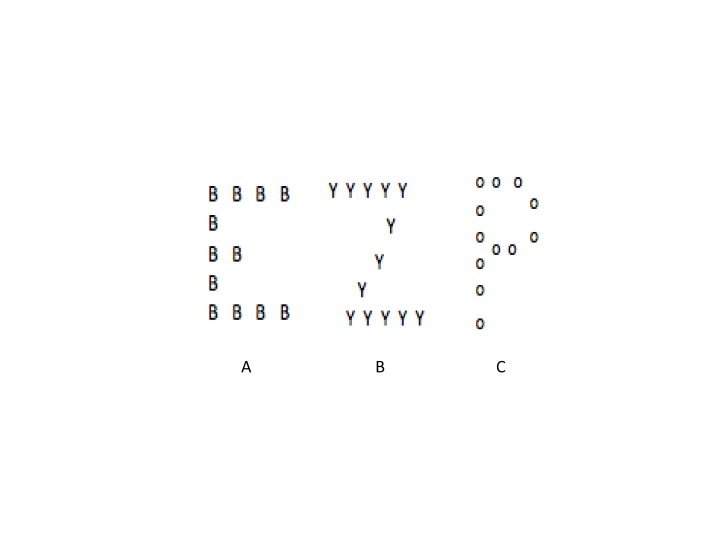
Examples of Navon stimuli. Panel A. Global stimulus is the letter *E*, and local stimulus is the letter *B*. Panel B. Global stimulus is the letter *Z*, and the local stimulus is the letter *Y*. Panel C. Global stimulus is the letter *P*, and the local stimulus is the letter *O*.

Navon (1977) developed a way to investigate, and bias, global and local visual processing applying a paradigm which used special stimuli, that became known as “Navon stimuli” (Figure 2). Navon used hierarchical letters, which consisted of a larger letter made up of many smaller letters. The letters could be read out in either a global form (i.e., the large letter) or in a local form (i.e., one of the small letters comprising the larger letter). Using this type of stimulus, Navon presented evidence to support the contention that global processing shows precedence over local processing; that is, global processing of a stimulus is faster and more automatic than local processing, which requires a slower and more effortful mechanism^1^. Further studies have consistently supported his results (DeLillo, Spinozzi, Palumbo, & Giustino, 2011; Fink et al., 1997; Hochstein & Ahissar, 2002; Paquet, 1992; Tanaka & Fujita, 2000; Wyer, 2010). Adding to this, Macrae and Lewis (2002) implemented Navon stimuli to *modulate* global and local processing before a face recognition task. Intending to prove that default face processing is generally biased toward the global level, it was found that face recognition was significantly enhanced when previously exposed to the global Navon condition, compared with the control condition. Furthermore, face recognition was significantly impaired when previously exposed to the local condition compared to the control condition. Such findings demonstrate that a *processing bias* can be *induced* by simple exposure to Navon stimuli (see also Perfect, Dennis, & Snell, 2007).

The purpose of the current study was to empirically test Day’s (1989) theory of perceptual compromise within the Müller-Lyer illusion. Initial exposure to Navon stimuli was hypothesised to create a bias toward global or local processing which would impact upon the magnitude of the Müller-Lyer illusion in a later task. A bias created toward global processing (by focusing attention on the large Navon letters) should enhance the Müller-Lyer illusion in comparison to a control group. A bias toward local processing (by directing attention toward small Navon letters) should weaken the Müller-Lyer illusion in comparison to a control group.

## Method

### Participants

This study comprised a convenience sample of 306 Monash University undergraduate students (*M*_age_ = 23.81, *SD*_age_ = 6.76; 76.8% female, 23.2% male) who participated voluntarily, in return for course credit. The research was approved by the Monash University Human Research Ethics Committee (MUHREC), and all participants gave written informed consent prior to participation, in accordance with the guidelines of MUHREC. Participants were randomly assigned into the three Navon treatment conditions: global, local, and control (no Navon exposure).

### Materials

Participants selected into the global and local conditions were asked to view a timed presentation of Navon stimuli (created using Microsoft Visual Basic©). The stimuli appeared at a size of 500 × 500 pixels on a 21-in. widescreen LCD monitor. Three hundred Navon stimuli were made for this study (see Figure 2) and were presented individually on screen for a period of 1 s, for a total presentation time of 5 min for the whole sequence. The effects of the processing level bias modulation were then examined using a second custom-written computer program, designed to test the magnitude of the Müller-Lyer illusion. The program was written in the Microsoft Visual Basic© programming language.

The parameters for the Müller-Lyer program were as follows: For each trial, participants were asked to match the apparent length of a line between arrowheads with an adjustable comparison line (see Panel C of Figure 1). The length of the adjustable stimulus could be modified by clicking on-screen buttons marked “shorter” or “longer.” Participants were asked to click these buttons until there was a perceptual match between the adjusted line and the length of the Müller-Lyer illusion. The adjustable comparison line was randomly set to either 100 or 200 pixels at the start of each trial to limit systematic instrumentation errors. The Müller-Lyer illusory stimulus was randomly set to between 80 and 250 pixels to prevent learning effects. The angle between the arrowhead lines was also varied on each trial, between 15 and 165°, in 15° increments (i.e., 15, 30, 45, 60, 75, 90, 105, 120, 135, 150, 165°). Fins at angles from 15 to 75° pointed inwards (fins-in), whereas fins at angles from 105 to 165° pointed outwards (fins-out). On a given trial, a fin angle was chosen at random from the 11 possibilities, with the restriction that each angle was used eight times across the experiment, and could not be presented twice in immediate succession. Thus, there were a total of 88 trials. The purpose of varying fin angles in this study was to observe the magnitude of the Müller-Lyer illusion over several different stimuli and to prevent the development of practice effects. After adjustment of the comparison line, the participant’s illusion magnitude was measured as the difference in length between the adjusted and illusory lines: “adjustment error.” Negative values would indicate adjustments too short, whereas positive values indicate adjustments too long.

### Procedure

Each participant was seated in a darkened room, 70 cm from a 21-in. PC computer monitor. Chair height was adjusted so that the participant’s eyes were level with the centre of the computer screen. Participants were randomly allocated to either local processing bias, global processing bias, or the control condition. Participants in the global condition were instructed to view the Navon presentation sequence, reading aloud the large letters, quickly and accurately. Those in the local condition were asked to read out the small letters. The experimenter monitored participants’ responses for compliance. After the 5 min Navon sequence these participants were then asked to complete the Müller-Lyer experiment. Participants allocated to the control condition commenced the Müller-Lyer portion of the experiment immediately.

## Results

**Figure 3.**
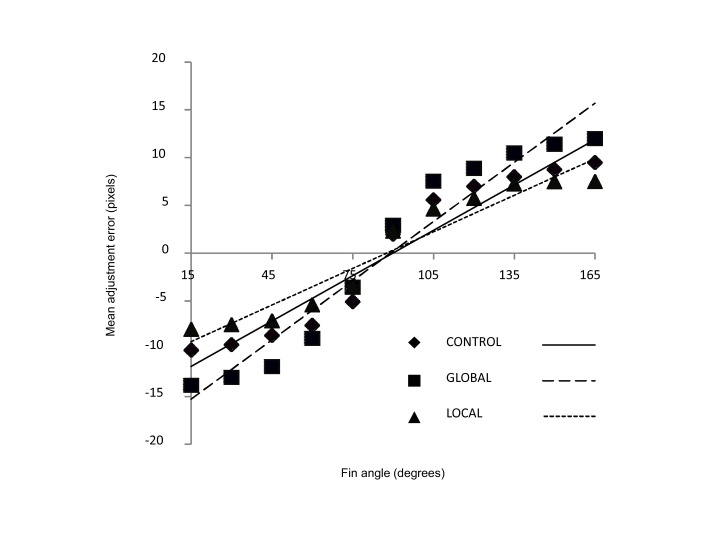
Mean adjustment error (MAE) for each of the Navon treatment conditions (global – squares, dashed line; local – triangles, dotted line; and control – triangles, solid line). Adjustment error is measured as the difference between the adjustable line and the presented stimulus, in pixels. For fins-in angles (15 to 75°), a negative MAE was obtained indicating that the illusory stimulus appeared shorter than in reality. For fins-out angles (90 to 165°), a positive MAE indicates that the illusory stimulus appeared longer. The gradient of the regression line equates to the magnitude of the illusion effect.

Mean adjustment error at each fin angle was calculated for each participant (i.e., a mean of any adjustment error across the eight trials at each fin angle). Mean adjustment error is plotted against fin angle for each condition in Figure 3. Negative error values indicate that comparison lines were adjusted to be smaller than the Müller-Lyer stimulus. Positive error values indicate that comparison line was adjusted to be larger than the Müller-Lyer stimulus. The regression relationship between adjustment error and fin angle can thus be considered to indicate the *intensity* of the illusion. The slope/gradient of the regression line between fin angle and adjustment error was therefore calculated for each participant. The gradient of the linear trend (*m*) was calculated as follows: *m* = (*y* - *c*)/*x*, where *c* is the *y*-axis intercept, *x*-axis is the fin angle, and *y*-axis is mean adjustment error. This data was transferred to IBM SPSS Statistics 20 for further analysis.

Inspection of Figure 3 indicates that overall, fins-in stimuli create negative adjustment errors: Participants perceive these stimuli to be shorter than they really are. On the other hand, fins-out stimuli create positive adjustment errors: Participants perceive these to be longer than they are. This relationship appears to follow a standard psychophysical sigmoid. The gradient of the relationship between fin angle and adjustment error (i.e., the strength of the illusion) was steeper overall for the global condition, compared with control, indicating a stronger illusion in this condition; whereas the gradient was shallower for the local condition, indicating a weaker illusion (see Table 1).

**Table 1.** Mean Gradient and Standard Deviation for Control, Local, and Global Groups

Group	*n*	MAE gradient	*SD*
Control	102	.158	0.068
Global	102	.206	0.078
Local	102	.127	0.063

*Note.* Mean adjustment error (MAE) gradient is the resulting regression slope of each condition’s mean adjustment error for each fin angle. The steeper the slope (higher value) indicates a greater mean adjustment error across all fin angles. Therefore, a higher slope value represents greater influence of the Müller-Lyer illusion.

These observations are supported by analysis of the gradient data in SPSS. ANOVA showed a significant between-subjects main effect of condition: global, local, control; *F*(2, 305) = 43.97, *p* < .001, η^2^ = .22. Homogeneity of variance and normality were confirmed by Levene’s statistic (*p* = .105) and the Shapiro-Wilk test (*p* = .845), respectively. Post-hoc comparisons using Tukey’s HSD confirmed that the global condition gradient was significantly steeper than both local and control, minimum *F*(1, 203) = 20.89, *p* < .001, indicting a stronger illusion in this group. The local condition produced a gradient which was significantly shallower than global or control, minimum *F*(1, 203) = 23.01, *p* < .001, indicating a weaker illusion. Our finding appears to support the suggestion that creating a bias toward either global or local processing will influence the strength of the visual illusion, according to Day’s (1989) hypothesis of perceptual compromise.

## Discussion

The purpose of this study was to test the potential for Day’s (1989) hypothesis of perceptual compromise to explain the estimation biases seen in the Müller-Lyer illusion. Day proposed that the presence of the illusion is due to a conflict between global cues and local cues. Exposure to Navon stimuli was used to create a bias in processing level toward either global or local, before testing the illusory strength of the Müller-Lyer illusion.

Supporting our interpretation of Day’s hypothesis, the strength of the Müller-Lyer illusion was significantly increased for participants in the global processing bias group, in comparison to those in the control condition; and it was significantly decreased for participants in the local processing bias group, in comparison to those in the control condition^2^. This finding sits well with the hypothesis that visual illusions such as Müller-Lyer are brought about by a tendency for the visual system to compromise between stimulus parts and their wholes. A strengthening of the illusion when biased toward global processing indicates that perceptual compromise is shifted toward the overall size of the figure. Conversely, a weakening of the illusion following bias toward local processing indicates a reduction in perceptual compromise and a restored ability to accurately judge local features.

In further support of these data, studies investigating cortical acti-vity during perception of the Müller-Lyer illusion have demonstrated that right-hemispheric visual regions, known to be global processing dominant, are more active than equivalent left-hemispheric regions, thought to be more active in local processing (e.g., Weidner, Boers, Mathiak, Dammers, & Fink, 2010; Weidner & Fink, 2007; see also Martens & Hübner, 2013). Knowing that our visual system is normally biased toward fast, global processing (Navon, 1977), it seems logical that such a pre-existing bias would influence our perception of the illusion, in terms similar to those suggested by Day (1989). Such neural-level findings, showing that lateral occipital, interior temporal, and dorsal visual stream regions contribute to the illusion, also strengthen the contention that higher-order visual areas must be involved (thus limiting theories which rely on low-level visual properties).

It must also be noted that the “assimilation theory” of Pressey (1967, 1971) bears resemblance to the ideas put forward by Day (1989). Pressey’s assimilation theory argues that the length of the central line is misperceived, since the visual system cannot successfully *isolate* local feature parts from global wholes (but see also Howe & Purves, 2005). Technically therefore, the end point of this theory is the same as Day predicts: A line with fins-out is seen as longer because the stimulus is, effectively, longer. Our Navon manipulation, when participants focus on the local letter features, may thus allow more effective featural isolation in the illusion, which would have the same outcome as reduced global compromise.

Furthermore, from these data it is not impossible to discount Gregory’s (1963) misapplied size constancy theory. For example, when a bias is formed toward the local features of the stimulus, this situation may reduce the likelihood that the figure would produce the three-dimensional global gestalt that - as Gregory suggests - is responsible for the impression of depth (and thus the application of size constancy) in the illusion. In effect, the manipulation of global and local bias may be modifying the extent to which the features of the figure are seen as distinct from one another, or as a whole.

It is clear from the plethora of theory and empirical data on the Müller-Lyer illusion and related visual phenomena that simple mechanistic explanations are unlikely to capture the full scope of their neural or cognitive basis. Further investigation is of course required to examine the effect described here in more detail. For example, eye tracking information would be very useful in determining any change in fixation or scan-pattern of the illusion following a Navon-like processing level manipulation.

## Footnotes

1 One can see, therefore, how such an arrangement may bring about Day’s perceptual compromise with regard to visual illusion: A pre-disposition to process global information before local detail will precipitate into the “conflicting cues” scenario.

2 Additionally, in line with previous research (e.g., Restle & Decker, 1977), as fin angle was increased it appeared that the strength of the Müller-Lyer illusion also increased.

## References

[R1] Ahluwalia A. (1978). An intra-cultural investigation of susceptibility to
“perspective” and “non-perspective” spatial
illusions.. British Journal of Psychology.

[R2] Bertulis A., Bulatov A. (2001). Distortions of length perception in human vision.. Biomedicine.

[R3] Day R. H., Vickers D., Smith P. L. (1989). Natural and artificial cues, perceptual compromise, and the basis
of veridical and illusory perception.. Human information processing: Measures, mechanisms, and models.

[R4] Day R. H. (1990). The Bourdon illusion in haptic space.. Perception & Psychophysics.

[R5] Day R. H., Jee F. M., Duffy F. M. (1989). Visual misalignment in arc and chevron figures.. Journal of Experimental Psychology: Human Perception and
Performance.

[R6] Day R. H., Kasperczyk R. T. (1985). Apparent displacement of lines and dots in a parallel-line
figure: A clue to the basis of the Poggendorff effect.. Perception & Psychophysics.

[R7] DeLillo C., Spinozzi G., Palumbo M., Giustino G. (2011). Attention allocation modulates the processing of hierarchical
visual patterns: A comparative analysis of the Capuchin monkeys (Cebus
apella) and humans.. Journal of Experimental Psychology: Animal Behavioral Processes.

[R8] Dewer R. E. (1967). Stimulus determinants of the magnitude of the Mueller-Lyer
illusion.. Perceptual and Motor Skills.

[R9] Fink G. R., Halligan P. W., Marshall J. C., Frith C. D., Frackowiak R. S., Dolan R. J. (1997). Neural mechanisms involved in the processing of global and local
aspects of hierarchically orga-nized visual stimuli.. Brain.

[R10] Gregory R. L. (1963). Distortion of visual space as inappropriate constancy
scaling.. Nature.

[R11] Hochstein S., Ahissar M. (2002). View from the top: Hierarchies and reverse hierarchies in the
visual system.. Neuron.

[R12] Howe C. Q., Purves D. (2005). The Müller-Lyer illusion explained by statistics of
image-source relationships.. Proceedings of the National Academy of Sciences of the United States of
America.

[R13] Lamy D., Segal H., Ruderman L. (2006). Grouping does not require attention.. Perception & Psychophysics.

[R14] Macrae C. N., Lewis H. L. (2002). Do I know you? Processing orientation and face
recognition.. Psychological Science.

[R15] Martens U., Hübner R. (2013). Functional hemispheric asymmetries of global/local processing
mirrored by the steady-state visual evoked potential.. Brain and Cognition.

[R16] McGraw K. O., Stanford J. (1994). The apparent distance of interior and exterior corners: A test of
Gregory’s misapplied size constancy explanation for the Mueller-Lyer
illusion.. The Journal of General Psychology.

[R17] Morgan M. J., Glennerster A. (1991). Efficiency of locating centres of dot clusters by human
observers.. Vision Research.

[R18] Müller-Lyer F. C. (1889). Optische Urteilstäuschungen. Archiv für Physiologie.

[R19] Navon D. (1977). Forest before the trees: The precedence of global features in
visual perception.. Cognitive Psychology.

[R20] Paquet L. (1992). Global and local processing in nonattended objects: A failure to
induce local processing dominance.. Journal of Experimental Psychology: Human Perception and
Performance.

[R21] Perfect T. J., Dennis I., Snell A. (2007). The effects of local and global processing orientation on
eyewitness identification performance.. Memory.

[R22] Pressey A. W. (1967). A theory of the Mueller-Lyer illusion.. Perceptual and Motor Skills.

[R23] Pressey A. W. (1971). An extension of assimilation theory to illusions of size, area,
and direction.. Perception & Psychophysics.

[R24] Pressey A. W., Martin N. S. (1990). The effects of varying fins in Müller-Lyer and Holding
illusions.. Psychological Research.

[R25] Restle F., Decker J. (1977). Size of the Mueller-Lyer illusion as a function of its
dimensions: Theory and data.. Perception & Psychophysics.

[R26] Robinson J. O. (1998). The psychology of visual illusion..

[R27] Tanaka H., Fujita I. (2000). Global and local processing of visual patterns in Macaque
monkeys.. NeuroReport.

[R28] Weidner R., Boers F., Mathiak K., Dammers J., Fink G. R. (2010). The temporal dynamics of the Müller-Lyer
illusion.. Cerebral Cortex.

[R29] Weidner R., Fink G. R. (2007). The neural mechanisms underlying the Müller-Lyer illusion
and its interaction with visuospatial judgements.. Cerebral Cortex.

[R30] Woloszyn M. R. (2010). Contrasting three popular explanations for the Müller-Lyer
illusion.. Current Research in Psychology.

[R31] Wyer R. S. (2010). Global and local processing: A clarification and
integration.. Psychological Inquiry.

